# Redefining Treatment Paradigms in Thyroid Eye Disease: Current and Future Therapeutic Strategies

**DOI:** 10.3390/jcm14155528

**Published:** 2025-08-06

**Authors:** Nicolò Ciarmatori, Flavia Quaranta Leoni, Francesco M. Quaranta Leoni

**Affiliations:** 1 Department of Translational Medicine, University of Ferrara, 44121 Ferrara, Italy; crmncl1@unife.it; 2 Department of Ophthalmology, Ospedali Privati Forlì “Villa Igea”, 47122 Forlì, Italy; 3 Ophthalmology Unit, Department of Experimental Medicine, University of Rome “Tor Vergata”, 00133 Rome, Italy; flavia.quarantaleoni@ptvonline.it; 4 Oftalmoplastica Roma, 00197 Rome, Italy; 5 Orbital and Adnexal Service, Tiberia Hospital, GVM Care & Research, 00137 Rome, Italy

**Keywords:** thyroid eye disease (TED), thyroid-associated orbitopathy (TAO), Graves’ orbitopathy, insulin-like growth factor-1 receptor (IGF-1R) inhibitors, teprotumumab, neonatal Fc receptor (FcRn) inhibitors

## Abstract

**Background:** Thyroid eye disease (TED) is a rare autoimmune orbital disorder predominantly associated with Graves’ disease. It is characterized by orbital inflammation, tissue remodeling, and potential visual morbidity. Conventional therapies, particularly systemic glucocorticoids, offer only partial symptomatic relief, failing to reverse chronic structural changes such as proptosis and diplopia, and are associated with substantial adverse effects. This review aims to synthesize recent developments in understandings of TED pathogenesis and to critically evaluate emerging therapeutic strategies. **Methods:** A systematic literature review was conducted using MEDLINE, Embase, and international clinical trial registries focusing on pivotal clinical trials and investigational therapies targeting core molecular pathways involved in TED. **Results:** Current evidence suggests that TED pathogenesis is primarily driven by the autoimmune activation of orbital fibroblasts (OFs) through thyrotropin receptor (TSH-R) and insulin-like growth factor-1 receptor (IGF-1R) signaling. Teprotumumab, a monoclonal IGF-1R inhibitor and the first therapy approved by the U.S. Food and Drug Administration for TED, has demonstrated substantial clinical benefit, including improvements in proptosis, diplopia, and quality of life. However, concerns remain regarding relapse rates and treatment-associated adverse events, particularly hearing impairment. Investigational therapies, including next-generation IGF-1R inhibitors, small-molecule antagonists, TSH-R inhibitors, neonatal Fc receptor (FcRn) blockers, cytokine-targeting agents, and gene-based interventions, are under development. These novel approaches aim to address both inflammatory and fibrotic components of TED. **Conclusions:** Teprotumumab has changed TED management but sustained control and toxicity reduction remain challenges. Future therapies should focus on targeted, mechanism-based, personalized approaches to improve long-term outcomes and patient quality of life.

## 1. Introduction

Thyroid eye disease (TED), also known as thyroid-associated orbitopathy (TAO) or Graves’ ophthalmopathy (GO), is a potentially sight-threatening autoimmune inflammatory disorder of the orbit, most commonly associated with Graves’ disease (GD). First described over a century ago, TED has evolved from a poorly understood complication of thyroid dysfunction to a distinct clinical entity with unique immunopathogenic mechanisms. It affects up to 40% of hyperthyroid patients and, although classically linked with hyperthyroidism, can also manifest in euthyroid or hypothyroid individuals [1,2].

With an estimated incidence of 5–20 per 100,000 person-years and a prevalence of approximately 0.25% in Western populations, TED is considered a rare disease [3]. Despite its rarity, it poses a significant socioeconomic burden as it often leads to significant physical disfigurement and psychosocial distress. Even patients with mild disease can experience substantial reductions in quality of life due to cosmetic concerns, chronic discomfort, and functional vision problems, leading to impaired daily functioning and productivity losses.

The disease typically follows a biphasic course, beginning with an active inflammatory phase lasting 1–3 years, characterized by signs such as eyelid retraction, chemosis, proptosis, and diplopia, and potentially progressing to dysthyroid optic neuropathy (DON), a sight-threatening complication. This is followed by a chronic, inactive phase during which residual fibrosis and tissue remodeling may persist.

Historically, TED management was primarily guided by clinical tools such as the Clinical Activity Score (CAS) and severity classifications like those from the European Group on Graves’ Orbitopathy (EUGOGO), which stratify disease as mild, moderate-to-severe, or sight-threatening. However, CAS remains limited by significant inter-observer variability, raising concerns about consistency in disease staging and treatment planning [4,5]. These limitations underscore the pressing need for more objective biomarkers and standardized assessment and treatment protocols.

Furthermore, traditional therapeutic approaches, including corticosteroids, orbital radiotherapy, and surgery, have been mostly supportive and symptomatic, limited by suboptimal efficacy in addressing chronic structural changes, and burdened by significant adverse effects. This highlights a critical unmet need for targeted mechanism-based therapeutic agents rather than symptom-based approaches.

Recently, the advent of targeted immunotherapies has represented a major paradigm shift in the management of TED, offering, for the first time, disease-modifying potential by directly interfering with the molecular pathways underlying inflammation and tissue remodeling. However, issues related to long-term results and relapse rates, safety profile, cost, and accessibility still limit their widespread adoption and remain under active investigation.

This review aims to provide an in-depth analysis of the evolving understanding of TED pathogenesis and to synthesize emerging evidence on the most recent therapeutic strategies. Analyzing these developments within the scope of established clinical frameworks underscores the persistent challenges and emerging opportunities in enhancing the quality of care for patients affected by this complex and debilitating condition.

## 2. Materials and Methods

A comprehensive literature search was conducted up to June 2025 using MEDLINE (via Ovid), Embase (via Ovid), and the US and EU Clinical Trials Registers, utilizing the following keywords: “Thyroid Eye Disease”, “Thyroid Orbitopathy”, “Graves’ Orbitopathy”, and “Graves’ Ophthalmopathy”. Boolean operators and MeSH terms were applied where appropriate.

The focus of the review was primarily on English language peer-reviewed articles, proof-of-concept studies, and publicly registered clinical trials addressing the pathophysiology of TED, pivotal clinical trials that have influenced current therapeutic strategies, and recent experimental and clinical investigations on novel therapies, including monoclonal antibodies and biologic agents. No restrictions concerning sex, age, follow-up time, setting, or number of participants were imposed.

Article selection was independently undertaken by the authors, involving title and abstract screening, followed by removal of duplicates and non-relevant publications. The reference lists of included articles were also manually reviewed to identify additional pertinent studies.

## 3. Results

### 3.1. Role of Glucocorticoids and Orbital Radiotherapy

Glucocorticoids (GCs) remain the first-line therapy for active TED in most European countries as per EUGOGO guidelines. However, the optimal treatment plan is still debated and protocols vary worldwide [6]. GCs modulate inflammation and immune responses via both direct and indirect genomic mechanisms, in addition to non-genomic pathways. This includes the induction of lipocortin synthesis, which subsequently inhibits leukotriene production, among other effects. Moreover, GCs mitigate the synthesis and release of cytokines, cell adhesion molecules, and glycosaminoglycans [7]. Consequently, GCs have been extensively utilized in the management of TED, although off-label, demonstrating the most substantial outcomes in immunosuppression when administered early during the active phase [8]. Intravenous methylprednisolone (IVMP) is favored over oral GCs for its higher bioavailability and faster onset [8].

However, recent evidence highlights the limited efficacy of GCs in addressing key clinical manifestations of TED. A meta-analysis reported a modest reduction in proptosis of 0.94 mm (95% CI: −1.57 to −0.32) at 12 weeks following IVMP therapy. Furthermore, a matching-adjusted indirect comparison found no statistically significant benefit of IVMP over a placebo in improving diplopia (odds ratio 2.69; 95% CI: 0.94–7.70) [9]. These findings underscore the limitations of GCs in targeting the fibrotic and structural changes underlying TED, leaving disfiguring symptoms such as proptosis and diplopia largely unresolved, with substantial implications for patients’ quality of life. While high-dose IVMP remains the mainstay in the management of DON, offering prompt visual improvement [10], its use is constrained by a significant adverse effect profile, including Cushingoid features, hypertension, hyperglycemia, psychiatric disturbances, and hepatotoxicity at cumulative doses exceeding 8 g [11].

Given the absence of robust, placebo-controlled trials supporting the usage of GCs, the necessity for novel pharmacological agents within the therapeutic arsenal for the treatment of TED is substantial and has consequently generated increasing interest in identifying new targets to disrupt the pathogenesis of TED.

Orbital radiotherapy (OR) is an established treatment for TED, primarily due to its anti-inflammatory effects and the radiosensitivity of infiltrating lymphocytes [12,13]. OR has demonstrated efficacy in the management of moderate-to-severe active TED, particularly in improving diplopia and ocular motility. A double-blind, sham-controlled RCT involving 60 patients confirmed the significant benefits of OR over sham treatment, while retrospective studies have reported superior outcomes when OR is combined with intravenous glucocorticoids compared to glucocorticoid monotherapy [14,15].

Longitudinal data further support the protective role of OR in disease progression: in a cohort of 351 patients with active TED, 17% of those treated with corticosteroids alone developed compressive optic neuropathy over 3.2 years, whereas no such cases were observed in patients receiving combined OR and corticosteroids, who also showed enhanced control of inflammation and ocular motility [16].

Combined with GCs in the active phase, OR reduces DON risk (0% versus 17%) [16]. Standard OR dosing is 20 Gy per orbit, administered as either 2 Gy over 10 days or 1 Gy over 20 days, with the latter showing better tolerability [17].

While generally well tolerated, OR may transiently exacerbate symptoms and is contraindicated in patients under 35 years of age or those with hypertensive or diabetic retinopathy due to potential long-term risks. Overall, OR remains an effective adjunctive therapy for TED, particularly during the active phase.

### 3.2. Role of Steroid-Sparing Agents

Nonsteroidal immunomodulatory agents have been explored as both adjunctive and standalone therapies in the treatment of moderate-to-severe active TED, with the goal of improving efficacy while minimizing the adverse effects associated with prolonged GC use.

**Mycophenolate mofetil (MMF).** MMF inhibits T and B cell proliferation through depletion of guanosine nucleotides. In a recent meta-analysis, MMF in combination with intravenous methylprednisolone (IVMP) demonstrated superior efficacy and tolerability compared to IVMP alone, with improvements in Clinical Activity Score (CAS) and reduced risk of adverse events [18]. The European Group on Graves’ Orbitopathy (EUGOGO) now recommends MMF plus IVMP as a first-line therapeutic option in moderate-to-severe active TED.

**Cyclosporine.** This calcineurin inhibitor suppresses IL-2 secretion and T cell activation. Several randomized controlled trials have shown that cyclosporine, when added to oral prednisolone, enhances therapeutic outcomes compared to prednisolone alone [6,19]. Based on these findings, EUGOGO recommends cyclosporine in combination with oral glucocorticoids as a second-line option for patients who fail to respond to IVGC monotherapy.

**Methotrexate (MTX).** MTX interferes with folate metabolism by inhibiting thymidylate synthase (TYMS) and dihydrofolate reductase (DHFR), thereby limiting the synthesis of nucleotides and the proliferation of inflammatory cells. Although robust RCTs are lacking, some studies have reported improvements in CAS; however, no significant effects on proptosis or diplopia have been demonstrated [20].

**Azathioprine.** Like MTX, azathioprine inhibits purine synthesis, impairing lymphocyte proliferation. Current evidence does not show significant long-term benefit over a placebo in TED, although it may play a role in reducing relapse rates following glucocorticoid tapering [21].

**Sirolimus (rapamycin).** Sirolimus is an mTOR pathway inhibitor with immunosuppressive and antiproliferative properties. Initial data were limited and often affected by prior interventions, but recent observational evidence suggests that sirolimus, when used in combination with IVMP, may offer superior outcomes in diplopia resolution compared to MMF from six months onward [22,23]. Its potential antifibrotic effect on extraocular muscles is of particular interest. The ongoing SIRGO trial is currently evaluating sirolimus as a first-line therapy for active TED [24].

**Rituximab (RTX).** RTX is a monoclonal antibody targeting CD20+ B cells. Early open-label and retrospective studies indicated reductions in CAS and TRAb levels, with some improvement in diplopia [25]. However, randomized trials have yielded inconsistent results, possibly due to heterogeneity in patient populations and disease stages. Consequently, RTX is generally reserved as a second-line treatment for corticosteroid-refractory cases.

**Statins.** Although primarily used for dyslipidemia, statins possess notable anti-inflammatory and immunomodulatory properties [26]. A recent systematic review reported that statin use was associated with reduced disease severity and slower progression of TED, suggesting a potential adjunctive role in its management [27].

### 3.3. Current Insights into the Pathogenesis of TED

TED’s multifactorial pathogenesis involves autoimmune activation of orbital fibroblasts (OFs) driving inflammation and extracellular matrix (ECM) remodeling. OFs exhibit phenotypic heterogeneity influenced by Thy-1 (CD90) expression: Thy-1^+^ OFs, in extraocular muscles, differentiate into myofibroblasts promoting fibrosis, while Thy-1^−^ OFs, in connective tissue, act as preadipocytes promoting adipogenesis [28,29].

These subsets interact with immune cells, sustaining inflammation, ECM expansion, and fibrosis [30,31]. ECM remodeling persists into the chronic phase, challenging the classic biphasic model and underscoring the need for therapies addressing both inflammation and fibrosis [32].

Table 1 and Table 2 summarize current and emerging targeted therapies for TED.

### 3.4. Role of TSH-R and Its Inhibitors

The thyroid-stimulating hormone receptor (TSH-R), a G-protein-coupled receptor, is a key autoantigen in GD and TED. Normally expressed on thyroid cells, TSH-R is aberrantly found on orbital preadipocyte fibroblasts and myofibroblasts in TED, promoting hyaluronic acid (HA) production and fibroblast proliferation [33].

In murine models, human TSH-R transfection induced orbital adipose and connective tissue changes mimicking TED [34]. During the active phase of TED, TSH-R expression is notably higher, and activation by TSH, thyrotropin-receptor antibodies (TRAb), and interleukin-6 (IL-6) triggers downstream signaling cascades that drive the characteristic extracellular matrix expansion and cellular proliferation [35]. These findings support TSH-R as a promising therapeutic target in TED.

#### 3.4.1. K1-70

K1-70 is a monoclonal antibody that inhibits TSH-R signaling. In a Phase I trial involving 18 stable TED patients, single intramuscular or intravenous doses led to complete CAS resolution and up to 8 mm proptosis reduction, with minimal adverse events (AEs) such as fatigue and diarrhea [36]. A Phase II Japanese randomized, double-blind, placebo-controlled trial is currently evaluating 50 mg and 100 mg doses in active TED to confirm efficacy and safety [37].

#### 3.4.2. Small-Molecule TSH-R Antagonists

Small-molecule TSH-R antagonists are emerging as promising oral treatments for TED, offering favorable pharmacokinetics and potential cost benefits. These drugs act as allosteric inhibitors, meaning they interfere with TSH-R signaling inside the cell rather than blocking hormone binding at the receptor surface. For example, ANTAG3 reduced TRAb-induced cAMP and pAKT activity, as well as hyaluronic acid production, in OFs from TED patients [38].

Similarly, s37a inhibited cAMP buildup in TSH-R-expressing human embryonic kidney (HEK) cells stimulated with sera from TED patients [39]. In addition, TSH-R-targeted siRNA improved both clinical symptoms and biochemical markers in a mouse model of Graves’ disease [40].

### 3.5. Role of IGF-1R and Its Inhibitors

Insulin-like growth factor 1 receptor (IGF-1R), a receptor tyrosine kinase (RTK), is overexpressed and hyperfunctional in OFs and lymphocytes in TED, where it regulates fibroblast proliferation, adipogenesis, and lymphocyte activation via MAPK/ERK and PI3K/AKT pathways [41].

Although early studies suggested the existence of stimulating autoantibodies against IGF-1R similar to those targeting the TSH-R, their pathogenic relevance remains uncertain. IGF-1R antibody levels show no positive correlation with TED severity and may inversely associate with the CAS, indicating a potential protective role [42].

Current data support TSH-R/IGF-1R crosstalk as the primary driver of IGF-1R activation in TED [43]. The therapeutic relevance of this axis is underscored by the effectiveness of IGF-1R antagonists in suppressing TSH-R-induced hyaluronic acid (HA) production and cell proliferation and shows therapeutic potential in TED and oncology models by promoting apoptosis and reducing proptosis [44].

#### 3.5.1. Teprotumumab

Teprotumumab (Tepezza), a fully human monoclonal antibody targeting IGF-1R, represents a significant advancement in TED therapy. Originally developed for oncology, it was repurposed after IGF-1R’s role in TED was identified [45]. Administered intravenously (10 mg/kg, then 20 mg/kg every 3 weeks for 8 doses) over 60–90 min, it showed efficacy in two pivotal RCTs: a Phase II trial by Smith et al. and the Phase III OPTIC trial, where 83% achieved ≥2 mm proptosis reduction compared to 10% on a placebo, with additional gains in diplopia and quality of life [46,47]. Following FDA approval in 2020, the 2022 OPTIC-X extension demonstrated sustained improvement in relapsing or non-responsive patients (89.2%) [48]. A subsequent RCT confirmed efficacy in chronic TED, leading to FDA indication extension to TED of any activity or duration [49].

Table 3 summarizes key clinical trials of teprotumumab in TED management.

Beyond regulatory milestones, further insights have emerged from imaging studies, real-world data, and subgroup analyses that deepen our understanding of teprotumumab’s mechanisms of action and clinical applications.

Imaging and real-world data confirmed orbital volume reductions [52,53] and DON improvement in up to 88% of cases [50], although relapse occurred in up to two-thirds within one year, suggesting some patients may require retreatment or combination with surgery [48,54,55]. Notably, a recent study found no significant association between the timing of post-teprotumumab orbital surgery and regression. However, delayed surgeries (≥180 days) were linked to greater disease activity and surgical burden [56].

Although teprotumumab has shown an acceptable safety profile, clinical trials and postmarketing surveillance highlighted an association with muscle spasms, hyperglycemia, inflammatory bowel disease, and hearing impairment, prompting routine audiometric evaluation [57].

Despite its transformative role in TED, the high cost and limited long-term data restrict its widespread use [58], although an upcoming Phase III trial of a subcutaneous formulation aims to improve convenience and adherence [59].

The 2022 ATA/ETA consensus recommends teprotumumab for patients with significant proptosis or diplopia, reflecting its established efficacy in moderate-to-severe active TED. In contrast, the EUGOGO guidelines currently consider teprotumumab a second-line option, citing the absence of head-to-head comparative data and the lack of European Medicines Agency (EMA) approval as of 2025 [4]. Teprotumumab is currently under regulatory review in Europe and Canada and has received approval in the United States, Australia, Brazil, Saudi Arabia, the United Arab Emirates, and Japan. The OPTIC-J trial has further confirmed its efficacy and safety in a Japanese cohort, supporting its potential for broader global implementation [51].

Overall, teprotumumab has significantly advanced TED management, particularly for proptosis and diplopia, though its optimal application requires careful patient selection, vigilant safety monitoring, and consideration of treatment cost and durability.

#### 3.5.2. IBI311

IBI311, a recombinant anti-IGF-1R mAB under review by China’s NMPA, follows the teprotumumab dosing schedule. In the Phase III RESTORE-1 trial, 85.8% of Chinese TED patients achieved CAS improvement and ≥2 mm proptosis reduction at week 24 versus 3.8% with a placebo (*p* < 0.0001). Although trial data regarding AEs are pending, the safety profile was reported favorable [60].

#### 3.5.3. Veligrotug (VRDN-001) and VRDN-003

Veligrotug (VRDN-001), a humanized anti-IGF-1R monoclonal antibody by Viridian Therapeutics, offers a shorter infusion schedule compared to teprotumumab, consisting of five 30-min intravenous infusions (10 mg/kg) every three weeks. Of the key clinical trials that are summarized in Table 4, two pivotal Phase III trials, THRIVE and THRIVE-2, have confirmed its efficacy and safety in active and chronic TED, respectively.

The THRIVE trial demonstrated that Veligrotug significantly improved outcomes in patients with active TED by week 15, with a proptosis responder rate (PRR) of 70% (64% placebo-adjusted). Diplopia resolved in 54% of treated patients (43% placebo-adjusted), and 64% achieved a CAS of 0 or 1 (46% placebo-adjusted). The safety profile was favorable, with no serious treatment-related adverse events; common side effects included muscle spasms (43%), headache (21%), and infusion reactions (17%). Hearing impairment occurred in 16% of treated patients versus 11% on a placebo (5.5% placebo-adjusted) [61].

In THRIVE-2, Veligrotug led to 2.34 mm proptosis reduction versus 0.46 mm with placebo, diplopia improvement in 56% versus 25%, and CAS ≤ 1 in 54% versus 24% (*p* < 0.01). Reported AEs were mild, including muscle spasms (36%), menstrual disorders (33%), and hearing impairment in 13% [62].

Veligrotug is currently under investigation in the STRIVE trial, a randomized, active-controlled study assessing efficacy, safety, and tolerability in TED patients irrespective of disease activity or duration [63]. Based on favorable Phase III outcomes, Viridian Therapeutics intends to submit a Biologics License Application (BLA) in the second half of 2025.

Furthermore, Viridian is advancing VRDN-003, a long-acting IGF-1R inhibitor intended for subcutaneous administration. Clinical regimens under evaluation include a 600 mg loading dose, followed by two 300 mg injections every eight weeks or five 300 mg injections every four weeks. VRDN-003 is currently being studied in the REVEAL-1 and REVEAL-2 Phase III trials for active and chronic TED, respectively, with results expected in the first half of 2026 [64].

#### 3.5.4. Lonigutamab

Lonigutamab, a subcutaneous anti-IGF-1R monoclonal antibody by Acelyrin Inc., Agoura Hills, CA, USA demonstrated promising results in a Phase I/II trial for active TED. Two regimens, four 40 mg injections every 3 weeks versus a 50 mg loading dose followed by 25 mg weekly for 11 weeks, were well tolerated without ototoxicity. The 40 mg group showed a 50% proptosis response, a 25% improvement in diplopia, and consistent reductions in CAS, compared to no improvements in the placebo group. The weekly regimen yielded higher responses: 67% proptosis, 40% diplopia resolution, and 83% achieving ≥2-point CAS reduction, with slightly milder adverse events. These results support subcutaneous IGF-1R inhibition as a safe and effective TED therapy with sustained response and potential for extended dosing [65].

#### 3.5.5. Linsitinib

Linsitinib is an oral dual inhibitor of IGF-1R and insulin receptor (IR) tyrosine kinase activity, disrupting PI3K/Akt and ERK signaling and TSH-R/IGF-1R crosstalk, which are central to TED pathogenesis. Preclinical murine models showed that Linsitinib inhibits OFs proliferation, hyaluronic acid secretion, and immune-driven tissue remodeling, while inducing apoptosis in IGF-1R/TSH-R-expressing cells [66,67].

In the Phase IIb LIDS trial involving 90 patients with active moderate-to-severe TED, linsitinib (150 mg twice daily) achieved a 52% proptosis responder rate at week 24, marking the first oral small molecule to demonstrate clinical efficacy in TED. Common adverse events included diarrhea, headache, nausea, fatigue, and elevated liver enzymes (~20), with no cases of hearing impairment or hyperglycemia reported [68].

A Phase III trial is planned for 2025 to evaluate long-term safety and efficacy in refractory or relapsing TED.

#### 3.5.6. KRIYA-586

Kriya-586 is an investigational gene therapy by Kriya Therapeutics that delivers an IGF-1R-blocking antibody via a single peribulbar injection using an adeno-associated virus (AAV) vector. This one-time treatment allows sustained antibody expression, potentially eliminating the need for repeated dosing and significantly improving quality of life.

Preclinical in vivo studies have demonstrated effective suppression of IGF-1R-mediated signaling, with efficacy comparable to that of teprotumumab. First-in-human trials are expected to start in late 2025 [69].

### 3.6. Role of FcRn and Its Inhibitors

The neonatal Fc receptor (FcRn) extends the half-life of IgGs by protecting them from degradation in acidic endosomes and recycling them back to the bloodstream, a mechanism that also preserves pathogenic autoantibodies in autoimmune diseases [70].

FcRn inhibitors competitively bind to FcRn, promoting the lysosomal degradation of IgG and thereby selectively reducing their levels without affecting albumin, immune cells, cytokines, complement activity, or other immunoglobulins. This lowers the risk of infection compared to broad-spectrum immunosuppressants.

Targeting FcRn has recently emerged as a promising therapeutic approach for TED, with various drugs currently under evaluation for long-term safety and efficacy in TED, as summarized in Table 5.

**Batoclimab (HBM9161)**, a human monoclonal antibody against FcRn, demonstrated a 64.8% reduction in serum IgG and a 56.7% decrease in anti-TSHR antibodies in the Phase IIa ASCEND-GO 1 trial involving patients with active moderate-to-severe TED, with predominantly mild, reversible adverse effects. However, the ASCEND-GO 2 trial was terminated early due to reversible hypercholesterolemia, raising safety concerns [71]. A Phase III trial is ongoing to further assess its efficacy and safety profile [72].

**Efgartigimod**, an engineered IgG1 Fc fragment with enhanced FcRn affinity, selectively reduces IgG while sparing IgM, IgA, and albumin. Originally approved by the FDA for myasthenia gravis, it has also shown benefits in other IgG-mediated diseases, including primary immune thrombocytopenia [75,76]. In TED, Efgartigimod is under investigation in the Phase III UplighTED trial, a randomized, double-masked, placebo-controlled study of patients with active moderate-to-severe disease, with a 2:1 randomization to drug or placebo, follow-up of up to 110 weeks, and an open-label extension. Preliminary data suggest a favorable safety profile, with mainly mild adverse events [73].

Viridian Therapeutics’ FcRn inhibitors, **VRDN-006** and **VRDN-008**, have shown promising preclinical results. VRDN-006 demonstrated similar efficacy and safety to efgartigimod in non-human primates, without inducing hypercholesterolemia. Phase I human trials are planned for 2025. VRDN-008 offers an extended half-life and more durable IgG reduction compared to efgartigimod in murine models [74].

### 3.7. Role of Cytokines

Cytokines including IL-6, IL-11, IL-17, tumor necrosis factor alfa (TNFα), and transforming growth factor beta (TGF-β) play well-documented roles in the pathogenesis of TED [30,77].

#### 3.7.1. IL-6

IL-6 is a key pro-inflammatory cytokine involved in chronic inflammation and autoimmune diseases, including TED. It signals through a hexameric complex composed of IL-6, IL-6R (both soluble and membrane-bound), and gp130, activating the JAK/STAT3, Ras/MAPK, and PI3K–Akt pathways to promote B and T cell responses [78,79].

**Tocilizumab**, a humanized monoclonal antibody targeting both soluble and membrane-bound IL-6R, prevents gp130 dimerization and downstream inflammatory signaling. Initially approved for rheumatoid arthritis, it has shown efficacy in corticosteroid-refractory TED in a small RCT (n = 32), where 86% of patients achieved CAS < 3, with a median proptosis reduction of 1.5 mm compared to no improvement in the placebo group [80]. Reported AEs included neutropenia and hypercholesterolemia. Observational studies and meta-analyses support its second-line use, though larger RCTs are needed [81,82]. The ongoing Phase II TOGO trial is comparing intravenous tocilizumab with methylprednisolone in moderate-to-severe TED [83].

**Satralizumab** is a subcutaneous anti-IL-6R IgG2 antibody with extended half-life and reduced dosing frequency. Initially approved for neuromyelitis optica spectrum disorder (NMOSD), it was developed through innovative antibody recycling technology, which extends its plasma half-life and facilitates subcutaneous administration. After being approved by the FDA in 2019 for the management of NMOSD, satralizumab is now being investigated in the context of TED. Currently, two global, Phase III, randomized, double-masked, placebo-controlled, multicenter studies, namely SatraGO-1 and SatraGO-2, are evaluating the drug’s safety and efficacy in adults with moderate-to-severe active and chronic inactive TED. In the initial six months of both studies, participants will receive subcutaneous injections every two weeks for the first three doses, followed by every four weeks for an additional five months (seven doses in total). The primary endpoint for these studies is the reduction in proptosis of at least 2 mm from baseline after the initial six months of treatment. This will be followed by a six-month follow-up period during which retreatment will be individualized based on proptosis response. The expected completion date for these studies is 2026 [84].

#### 3.7.2. IL-11

IL-11 contributes to the development of fibrosis and inflammation in TED, and its parallel targeting in pulmonary fibrosis further underscores its potential as a therapeutic target in TED.

**LASN01**, a fully human monoclonal antibody targeting IL-11R, inhibits IL-11 signaling to reduce orbital fibrosis, inflammation, and remodeling, with preclinical models showing improvements in proptosis, CAS, and ocular motility. LASN01 is currently being evaluated intravenously in a Phase II trial, with CAS and proptosis reduction as primary endpoints [85].

#### 3.7.3. IL-17

IL-17, mainly secreted by Th17 cells, promotes inflammation, fibrosis, and adipogenesis in TED via IL17R signaling in OFs. Elevated IL-17 levels correlate with higher CAS, and IGF-1R activity amplifies Th17-mediated orbital remodeling [86,87].

**Vunakizumab (SHR-1314)**, a subcutaneously administered anti-IL-17A IgG1/κ antibody used in the treatment of psoriasis and ankylosing spondylitis, entered a TED trial but was discontinued early for commercial reasons [88].

**Secukinumab**, another anti-IL-17A IgG1κ monoclonal antibody, was evaluated in the Phase III ORBIT trial for the treatment of moderate-to-severe TED, which was halted early due to low efficacy despite acceptable safety [89].

## 4. Conclusions

TED is a debilitating, potentially sight-threatening autoimmune disorder that affects thousands annually. Historically, glucocorticoids have constituted the cornerstone of TED management and continue to serve as first-line therapy in many settings. While effective in mitigating orbital inflammation, glucocorticoids are limited in their capacity to address key manifestations such as proptosis and diplopia, and their long-term use is associated with considerable systemic toxicity. These limitations underscore the need for disease-modifying strategies that extend beyond symptomatic control.

Advances in the pathophysiological understanding of TED have catalyzed the development of precision-targeted therapies. Clinical trials and meta-analyses have shown that teprotumumab significantly reduces proptosis and diplopia, outperforming glucocorticoids in several domains, although direct head-to-head comparisons are still lacking [9,90]. EUGOGO management guidelines offer a structured, evidence-based framework for TED evaluation and treatment, and recommend a staged, activity- and severity-based approach, incorporating the CAS and risk stratification to guide therapeutic decision-making [6]. Although high-dose intravenous glucocorticoids remain the standard for moderate-to-severe active disease, EUGOGO acknowledges the shortcomings of conventional immunosuppression and endorses the incorporation of targeted therapies into the evolving treatment paradigm [91].

Beyond IGF-1R blockade, additional therapeutic targets are under investigation. TSH-R inhibitors aim to arrest autoimmune activation at its origin by blocking ligand–receptor interactions on orbital fibroblasts [36,37,38]. Cytokine inhibitors, particularly those targeting interleukins IL-6, IL-11, and IL-17, offer promise in modulating the inflammatory processes that drive TED. Elevated IL-6 levels correlate with disease activity, and IL-6 receptor blockade (e.g., tocilizumab) has demonstrated efficacy in reducing CAS, proptosis, and diplopia, particularly in glucocorticoid-refractory cases [82,92,93]. Emerging treatments such as statins and FcRn antagonists are also under active investigation. Statins, due to their immunomodulatory and anti-inflammatory properties, have been associated with decreased incidence and severity of TED in observational studies, likely through modulation of T-cell responses and fibroblast activity [94]. FcRn antagonists, by inhibiting IgG recycling, promote degradation of pathogenic thyroid-stimulating immunoglobulins, thereby reducing autoimmune stimulation [71,72,73].

Despite these advances, TED remains a clinically heterogeneous condition, characterized by variability in disease trajectory and therapeutic response. The future of TED management is likely to emphasize individualized, multimodal strategies. Integration of clinical, radiologic, serological, and molecular markers—such as TSH-R antibody titers, cytokine profiles, and fibroblast phenotypes—may enhance patient stratification and inform therapy selection, advancing the field toward a precision medicine framework.

As novel agents become available, combinatorial regimens—for example, concurrent IGF-1R and TSH-R inhibition or the addition of FcRn antagonists in relapsing cases—may enhance efficacy while mitigating toxicity. The development of long-acting and subcutaneous formulations (e.g., efgartigimod, lonigutamab) also holds potential for improving adherence and patient convenience. Nonetheless, high costs and limited global access to these biologics present substantial barriers. Ongoing cost-effectiveness analyses will be critical to inform reimbursement decisions and ensure equitable access. In parallel, the accumulation of real-world data and long-term safety profiles will be essential for broader clinical integration.

Clinicians should incorporate these novel therapies within established guidelines, including those from EUGOGO, ATA, and ETA. Targeted therapies may be particularly beneficial in patients with significant proptosis or recurrent disease, complementing traditional immunosuppression and surgical interventions. Multimodal approaches that combine immunomodulatory therapy with appropriately timed surgical management remain essential.

Surgical interventions—orbital decompression, strabismus correction, and eyelid procedures—continue to play a vital role in restoring function and appearance in the chronic or inactive phase. Their timing and extent should be guided by inflammatory activity and response to medical therapy [95]. Notably, early surgical planning in patients receiving agents such as teprotumumab may reduce fibrosis, procedural complexity, and enhance functional outcomes [96,97].

Ultimately, the evolution of TED management toward precision medicine will require not only therapeutic innovation but also standardized predictive tools and outcome metrics to guide treatment selection and monitor response. Addressing the economic and accessibility challenges in parallel with ongoing research will be crucial to optimizing long-term outcomes and quality of life in patients affected by this complex disorder.

## Figures and Tables

**Table 1:** Current and emerging IGF-1R-targeted therapies for TED.

Drug	Developer	Drug Type	Initially Developed for	Cohort and Clinical Trial Phase in TED	Dosing Regimen	Most Effective on	Side Effects in TED
**Teprotumumab** **(TEPEZZA^®^,** **2020 FDA** **Approval)**	Horizon/ Amgen, Thousand Oaks, CA, USA	Fully human mAb	Solid cancer	Active and Chronic TED (FDA approved/Phase IV)	Eight Q3W IV inj. (1st: 10 mg/kg, then 20 mg/kg)	CAS Proptosis Diplopia	Hearing Impairment, Hyperglycemia, Muscle Spasms, Nausea/Diarrhea, Teratogenicity
**IBI311**	Innovent, Mountain View, CA, USA	Recombinant mAb	TED	Active TED (Phase IIb)	Eight Q3W IV inj. (1st: 10 mg/kg, then 20 mg/kg)	CAS Proptosis	Not Yet Reported, Expected Similar to Teprotumumab
**Veligrotug (VRDN-001)**	Viridian Therapeutics, Waltham, MA, USA	Humanized mAb	TED	Active and Chronic TED (Phase III)	Five Q3W IV inj. (10 mg/kg)	CAS Proptosis Diplopia	Muscle Spasms, Headache, Mild Hearing Issues
**VRDN-003**	Viridian Therapeutics, Waltham, MA, USA	Next-gen, half-life extended Humanized mAb	TED	Active and Chronic TED (Phase III)	Loading dose (600 mg) + Two Q8W or Five Q4W SC inj. (300 mg)	Not yet reported	Not Reported, Expected Similar to Veligrotug
**Lonigutamab**	ValenzaBIO, Bethesda, MD, USA/Acelyrin Inc., Agoura Hills, CA, USA	Humanized mAb	TED	Active TED (Phase Ib)	Four Q3W (40 mg) or Twelve QW SC inj. (1st 50 mg, then 25 mg)	CAS Proptosis Diplopia	Muscle Spasms, Headache, No Hearing Impairment
**Linsitinib**	Sling Therapeutics, Ann Arbor, MI, USA	Small molecule	Cancer	Active TED (Phase III)	75 mg or 150 mg tablets BID for 24 weeks	Proptosis	Diarrhea, Headache, Nausea, Fatigue, Elevated Liver Enzymes
**Kriya-586**	Kriya Therapeutics, Morrisville, NC, USA	AAV Gene Therapy	TED	Not specified (Phase I)	Single peribulbar injection	Not yet reported	Not Yet Reported

**Table 2:** Other current and emerging targeted therapies for TED.

Target	Drug	Developer	Initially Developed for	Cohort and Clinical Trial Phase in TED	Dosing Regimen	Most Effective on	Side Effects in TED
TSH-R	**K1-70**	RSR Ltd., Lancashire, UK	Thyroid Cancer	Active TED (Phase I)	Single dose 25 mg IM inj or Single dose 50/150 mg IV inj	CAS Proptosis	Fatigue, Diarrhea
FcRn	**Batoclimab**	Immunovant, New York, NY, USA	MG	Active TED (Phase III)	Six QW SC inj. (2 × 680 mg + 4 × 340 mg)	Lower TRAB CAS	Hypercholesterolemia, Hypoalbuminemia, Headache
	**Efgartigimod**	Argenx, Amsterdam, The Netherlands	MG	Active TED (Phase III)	Twentyfour QW SC inj. (1 g prefilled syringe)	Not yet reported	Not Yet Reported
IL6R	**Tocilizumab**	Hoffmann- La Roche, Basel, Switzerland	Rheumatoid Arthritis	Active TED (Phase II)	Four Q4W IV inj. (8 mg/kg)	CAS Proptosis (low)	Mild AEs, Neutropenia, Hypercholesterolemia
	**Satralizumab**	Hoffmann- La Roche	NMOSD	Active and Chronic TED (Phase III)	Three Q2W + Five Q4W SC inj. (120 mg)	Not yet reported	Not Yet Reported
	**Pacibekitug**	Tourmaline Bio, Inc., New York, NY, USA	ASCVD	Active TED (Phase IIb)	Three Q8W SC inj. (20 mg or 50 mg)	Not yet reported	Not Yet Reported
IL11R	**LASN01**	Lassen Therapeutics, Inc., San Diego, CA, USA	Pulmonary fibrosis	Active TED (Phase II)	Four Q4W IV inj. (multiple ascending dose)	Not yet reported	Not Yet Reported
IL17R	**Vunakizumab**	Suzhou Suncadia Biopharmaceuticals Co., Ltd., Beijing, China	Anti-rheumatic and Psoriasis	Active TED (Phase II— terminated early)	SC inj. (Details not reported)	Not reported	Not Reported
	**Secukinumab**	Novartis, Basel, Switzerland	Anti-rheumatic and Psoriasis	Active TED (Phase III— terminated early)	Four QW inj. + Two Q4W SC inj. (300 mg each)	Primary endpoint not met	Not Reported

**Table 3:** Key clinical trials of Teprotumumab.

Study	Study Population	Follow-Up Duration	Primary Endpoint	Key Findings	Main Adverse Events
**Smith et al., 2017 [46]**	RCT on moderate-to-severe active TED; average CAS ≥ 4 and disease duration < 9 months	24 weeks	Proptosis reduction ≥ 2 mm	Proptosis response: 71% vs. 20% CAS 0–1: 59% vs. 21%	Hyperglycemia in Diabetic Patients
**Douglas et al., 2020** **(OPTIC) [47]**	RCT on moderate-to-severe active TED; average CAS ≥ 4 and disease duration 6.3 months	24 weeks	Proptosis reduction ≥ 2 mm	Proptosis response: 83% vs. 10% CAS 0–1: 59% vs. 21% Diplopia response: 68% vs. 29% GO-QOL Score Improvement: 13.79 vs. 4.43	Muscle Spasms (25%) Nausea (17%) Alopecia (13%) Diarrhea (13%) Hearing Impairment (10%) Hyperglycemia (8%)
**Sears et al., 2021 [50]**	Retrospective observational case series	15 weeks	Unspecified	BCVA improvement: 0.87 logMAR Proptosis reduction: 4.7 mm CAS improvement: 5.25 points Diplopia improvement: 0.75 points RAPD resolution/improvement: 100% Color normalization/improvement: 100%	Not Reported
**Douglas et al., 2022** **(OPTIC-X) [48]**	Open-label extension study on non-responders or relapsed from OPTIC	48 weeks	Proptosis reduction ≥ 2 mm	Proptosis response: 89% CAS 0–1: 65.6% Diplopia response: 63% GO-QOL Score Improvement: 11.7 Proptosis recurrence at 6 m: 26%	Similar Profile to OPTIC
**Douglas et al., 2024 [49]**	RCT on inactive/Chronic TED; average CAS ≤ 1 and disease duration 2–10 years	24 weeks	Proptosis Reduction ≥ 2 mm	Proptosis response: 61.9% vs. 25% Diplopia Response not significant (study not powered enough)	Muscle Spasms (41.5%) Hearing Impairment (22%) Hyperglycemia (14.6%)
**Hiromatsu et al., 2025 (OPTIC-J) [51]**	RCT on Japanese patients with moderate-to-severe active TED; average CAS ≥ 3 and disease duration < 9 months	24 weeks	Proptosis reduction ≥ 2 mm	Proptosis improvement: 89% vs. 11% CAS 0–1: 59% vs. 22% Diplopia improvement: 64% vs. 45% (not significant)	Hyperglycemia (22%) Hearing Impairment (15%)

**Table 4:** Key clinical trials of Veligrotug.

Study	Study Population	Follow-Up Duration	Primary Endpoint	Key Findings	Main Adverse Events
**THRIVE [61]** **(2024)**	RCT on moderate-to-severe active TED; average CAS ≥ 4 and disease duration < 8 months	15 weeks	Proptosis reduction ≥ 2 mm	Proptosis response: 70% vs. 5% CAS 0–1: 64% vs. 18% Diplopia response: 63% vs. 20% Diplopia complete resolution: 54% vs. 12%	Muscle spasms (43%) Headache (21%) Hearing impairment (16%) Hyperglycemia (15%)
**THRIVE-2 [62]** **(2024)**	RCT on chronic TED; average CAS < 3 and disease duration > 5 years	15 weeks for primary endpoint, extended up to 52 weeks	Proptosis reduction ≥ 2 mm	Proptosis response: 56% vs. 8% CAS 0–1: 54% vs. 24% Diplopia response: 56% vs. 25% Diplopia complete resolution: 32% vs. 14%	Muscle spasms (6%) Menstrual disorders (33%) Headache (13%) Hearing impairment (13%) Diarrhea (10%) Hyperglycemia (5%)
**STRIVE** **(ongoing) [63]**	RCT on TED of any severity or duration	52 weeks	Incidence of treatment-emergent adverse events (TEAEs)	Expected late 2025	Not yet reported

**Table 5:** Anti-FcRn drugs in clinical development for TED.

Drug	Study	Population Tested	Follow-Up Duration	Treatment Regimen	Primary Endpoint	Key Findings	Main Adverse Events
**Batoclimab (HBM9161)**	ASCEND-GO 1 (Phase IIa) [71]	RCT on moderate-to-severe active TED	6 weeks	5 QW SC injections (680 mg 1st and 2nd, then 340 mg)	Changes in serum levels of anti-TSH-R Abs and total IgG	Significant reductions in serum IgG (64.8%) and anti-TSHR antibodies (56.7%)	Mild Reversible Hypoalbuminemia, Headache
**Batoclimab (HBM9161)**	ASCEND-GO 2 (Phase IIb) [71]	RCT on moderate-to-severe active TED	13 weeks	12 QW SC injections, various doses tested (680 mg, 340 mg, 255 mg)	Proptosis reduction ≥ 2 mm	Despite early termination due to unexpected increases in cholesterol levels, significant reductions in pathogenic antibodies Were observed	Reversible Increase in Serum Cholesterol Levels
**Batoclimab (HBM9161)**	NCT05517421 (Phase III) [72]	RCT on moderate-to-severe active TED	24 weeks	24 QW SC injections (twelve 680 mg inj. + twelve 340 mg inj.)	Incidence of treatment-emergent adverse events (TEAEs)	Expected in 2027	Not Yet Reported
**Efgartigimod**	UplighTED (Phase III) [73]	RCT on moderate-to-severe active TED	24 weeks for primary endpoint; extended up to 110 weeks	24 QW SC injections (1 g prefilled syringe)	Proptosis reduction ≥ 2 mm	Expected in 2027 (75–90% IgG reduction in preclinical murine models)	No Impact on IgM, IgA, Cholesterol or Albumin in Other Conditions Tested
**VRND-006**	Phase I [74]	Healthy volunteers	Not yet reported	Not yet reported	Not yet reported	Expected late 2025 Similar to Efgartigimod in preclinical trials.	Safety Profile Similar to Efgartigimod in Preclinical Trials

## Data Availability

Not applicable.

## Abbreviations

The following abbreviations are used in this manuscript:

TED	Thyroid Eye Disease
TAO	Thyroid-Associated Orbitopathy
GO	Graves’ Ophthalmopathy
GD	Graves’ Disease
DON	Dysthyroid Optic Neuropathy
CAS	Clinical Activity Score
GC	Glucocorticoid
IV	Intravenous
IVMP	Intravenous Methylprednisolone
SC	Subcutaneous
OR	Orbital Radiotherapy
MMF	Mycophenolate Mofetil
MTX	Methotrexate
RTX	Rituximab
TRAb	Thyrotropin Receptor Antibodies
RCT	Randomized Controlled Trial
ECM	Extracellular Matrix
OF	Orbital Fibroblast
THS-R	Thyroid-Stimulating Hormone Receptor
HA	Hyaluronic Acid
TSH	Thyroid-Stimulating Hormone
AE	Adverse Event
HEK	Human Embryonic Kidney
IGF-1	Insulin-like Growth Factor 1
IGF-1R	Insulin-like Growth Factor 1 Receptor
RTK	Receptor Tyrosine Kinase
FDA	Food and Drug Administration
EMA	European Medicine Agency
ATA	American Thyroid Association
ETA	European Thyroid Association
EUGOGO	European Group on Graves’ Orbitopathy
mAB	Monoclonal Antibody
NMPA	National Medical Products Administration
IR	Insulin Receptor
FcRn	Neonatal Fragment Crystallizable Receptor
TGF-ß	Transforming Growth Factor Beta
PRR	Proptosis Responder Rate
